# Human CAP cells represent a novel source for functional, miRNA-loaded exosome production

**DOI:** 10.1371/journal.pone.0221679

**Published:** 2019-08-28

**Authors:** Nikolas Zeh, Helga Schneider, Sven Mathias, Nadja Raab, Michael Kleemann, Sabine Schmidt-Hertel, Benjamin Weis, Silke Wissing, Nikola Strempel, René Handrick, Kerstin Otte

**Affiliations:** 1 Institute of Applied Biotechnology, University of Applied Sciences Biberach, Biberach, Germany; 2 CEVEC Pharmaceuticals GmbH, Köln, Germany; Virginia Commonwealth University, UNITED STATES

## Abstract

Exosomes represent a promising delivery tool for nucleic acid-based pharmaceuticals. They are highly suitable for transporting therapeutic miRNAs to tumor cells, due to their natural membrane components. Further, exosomes are capable of effectively protecting nucleic acids against ribonucleases and enable the delivery of their content through cell membranes. However, no suitable production host for miRNA containing exosomes of non-tumorigenic origin has yet been identified. In this study we engineered an immortalised human amniocyte cell line (CAP^**®**^ cells), whose exosomes were enriched and characterised. The cell line modifications not only enabled the production of GFP-labelled but also pro-apoptotic miRNA containing exosomes without negative influence on host cell growth. Furthermore, we demonstrated that pro-apoptotic miRNA containing CAP exosomes are taken up by ovarian cancer cells. Strikingly, delivery of functional exosomal miRNA led to downregulation of several reported target genes in the treated tumor cells. In summary, we revealed CAP cells of non-tumorigenic origin as a novel and efficient exosome production host with the potential to produce functional miRNA-loaded exosomes.

## Introduction

Exosomes are small membrane vesicles of 50–150 nm in size, which originate from the endosomal pathway by fusion of intracellular multivesicular bodies (MVB) with the plasma membrane and are thus released into the extracellular space [1,2]. Many different cell types, especially T-cells, dendritic cells and tumor cells release large amounts of extracellular vesicles (EVs) like exosomes, which are involved in various biological functions including regulation of immune responses, antigen presentation, tumor proliferation and intercellular communication [3–8]. In order to exert their functions, exosomes can fuse with the plasma membrane of a recipient cell to release their content into the cytosol, undergo endocytosis or bind to membrane receptors to activate signalling pathways [9,10]. Depending on their origin, exosomes contain specific profiles of cellular proteins, signaling proteins and peptides, microRNAs (miRNAs), messenger RNAs (mRNAs) and lipids [10,11]. Especially small non-coding regulatory RNAs like miRNAs are frequently detected in exosomes of nearly all cell types.

miRNAs are versatile modulators of gene expression and can downregulate numerous genes post-transcriptionally. A single miRNA is able to affect the expression of hundreds of target mRNAs, exerting significant influence in all pathways [12,13]. Especially in the context of cancer, miRNAs play a key role by deregulation of the miRNA balance observed in several tumor cell lines [14,15]. Thereby, a number of miRNAs showed first promising results as biomarkers or nucleic acid-based therapeutics to specifically induce apoptosis in tumor cells [16–19]. Most challenging in this context is the application of pro-apoptotic miRNAs *in vivo*, as these exhibit a very short half-life due to highly abundant ribonucleases [20]. Additionally, miRNAs possess low ability to penetrate biological membranes [21]. To circumvent these challenges, several approaches were developed including chemical modification, nanoscale coordination polymers or packaging of miRNA into synthetic liposomes [22–24]. Nevertheless, these modifications cause additional limitations as synthetic vehicles can trigger immune reactions [25]. A very promising alternative approach might be the use of extracellular vesicles such as exosomes to deliver miRNAs into tumor cells. Since exosomes consist of natural membranes, are capable of transporting nucleic acids in a stable manner and are taken up by target cells through fusion or endocytosis, they represent optimal carriers of miRNA-based therapeutics [1,26,27].

The choice of an optimal donor cell type for miRNA-loaded exosomes is one of the crucial prerequisites for developing an efficient and save exosome-based drug delivery system. A variety of cell types have been used experimentally to secrete exosomes, including immature dendritic cells and mesenchymal stem cells, as well as model cell lines such as HEK293T, HeLa and murine melanoma cell lines [28,29]. Immortalized cell lines offer particular advantages for the generation of exosomes as they infinitely provide cells for exosome production, display high proliferative rates and can easily be genetically modified. However, most immortalized cell lines are of tumorous origin and may carry harmful or even carcinogenic constituents, which may hamper their use as donor cells for the supply of therapeutic exosomes [30,31]. To circumvent these disadvantages, we aimed at evaluating an alternative human source for exosomes of non-tumor origin. “CEVEC’s Amniocyte Production cell line” (CAP) was originally derived from primary human amniocytes and immortalized using modified adenoviral functions [32,33]. CAP cells hold a completely documented history, which make them interesting hosts for recombinant protein and viral vector production [32–35]. Recently, we have shown that CAP cells can be engineered successfully to overexpress miRNAs for the optimization of biopharmaceutical production [36].

The current study further exploits the potential of engineered CAP cells to serve as alternative exosome production host. Since miRNA-744 and miRNA-493 were previously shown to exert pro-apoptotic effects in various ovarian cancer cell lines, we aimed at pre-loading these therapeutic miRNAs into CAP exosomes using genetic engineering and analyzed their molecular cargo and potential to functionally deliver these miRNAs into target cells of ovarian cancer origin.

## Results

### Generation of CAP cell lines for exosome tracking and delivery

In this study, an immortalized human amniocyte cell line (CAP cells) was used for engineering and producing exosomes. For the evaluation of CAP cells as production hosts for exosomes, the parental CAP cell line was engineered by using the pStbl-bsd-CMV-CD63-tGFP vector to overexpress the tetraspanin CD63 fused to GFP. This allowed for tracing of produced exosomes while validating exosome isolation procedures using flow cytometry analysis, as well as tracing their delivery to recipient cells using fluorescence microscopy. In order to achieve pre-loading of exosomes with miRNAs by cellular pathways, the resulting stable CAP-CD63-GFP cells were additionally engineered to overexpress either miRNA-493 or miRNA-744. The respective genomic human pre-miRNA sequences were PCR amplified from isolated CAP cell genomic DNA, subcloned into the miRNASelect^™^ pEGP-miR cloning and expression vector and used for the generation of CAP cell lines overexpressing the respective miRNA. Table 1 summarizes the obtained engineered CAP cell pools including miRNA-overexpression and control cell pools.

**Table 1:** Engineered CAP^®^ cell lines for the analysis of exosomes.

Name (cell line)	Expression vectors	Overexpressed genes
CAP	-	-
CAP-CD63-tGFP	pStbl-CMV-bsd-CD63-tGFP-vector	CD63-tGFP
CAP-pStbl-empty	pStbl-CMV-bsd-Null-vector	-
CAP-CD63-tGFP-Null	pStbl-CMV-bsd-CD63-tGFP-vector pEGP-CMV-Null-vector	CD63-tGFP
CAP-pStbl-empty-Null	pStbl-CMV-bsd-Null-vector pEGP-CMV-Null-vector	-
CAP-CD63-tGFP-miRNA-493	pStbl-CMV-bsd-CD63-tGFP-vector pEGP-CMV-hsa-miRNA-493-vector	CD63-tGFP hsa-miRNA-493
CAP-CD63-tGFP-miRNA-744	pStbl-CMV-bsd-CD63-tGFP-vector pEGP-CMV-hsa-miRNA-744-vector	CD63-tGFP hsa-miRNA-744

### Morphological and biochemical analysis verifies enrichment of CAP exosomes

**Fig 1:**
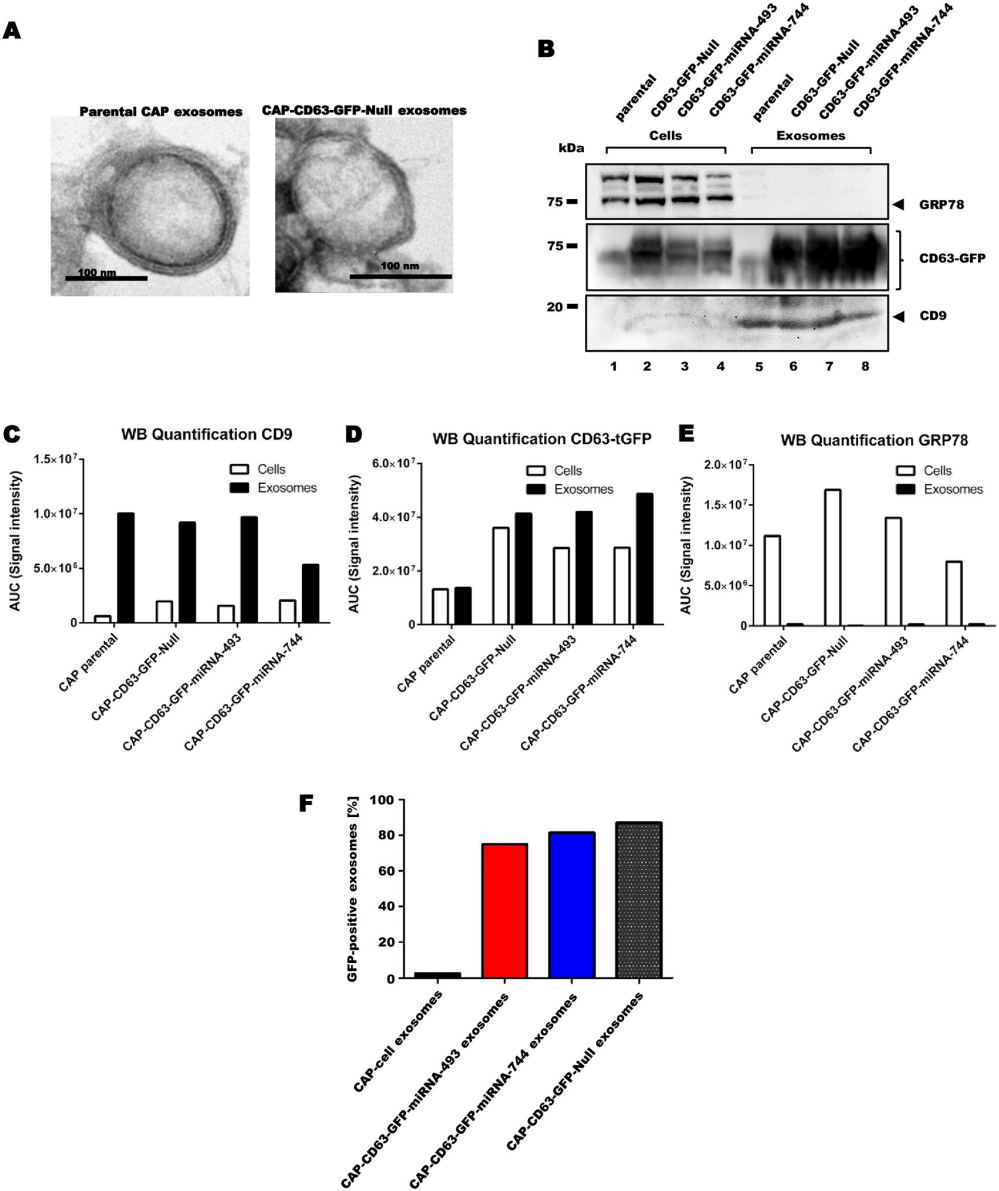
Morphological and biochemical analysis of isolated CAP exosomes. (**A**) Transmission electron microscopy pictures of parental (left) and modified (right) CAP exsomes. Scale bar represents 100 nm. (**B**) Western blot of 30 μg cellular and exosomal protein lysates per lane. Blotting performed on GRP78 (upper panel), turbo-GFP (middle panel) and CD9 protein (lower panel). Lane 1–4 represent cellular lysates, while lanes 5–8 depict exosomal lysate. (**C**) Quantification of Western blot Fig 1 B for CD9 signal. (**D**) Quantification of Western blot Fig 1 B for tGFP signal. (**E**) Quantification of Western blot Fig 1 B for GRP78 signal. (**F**) Flow cytometry analysis of GFP-positive exosomes isolated from parental and modified CAP cells.

For the analysis of CAP exosomes on morphological and molecular level, exosomes were concentrated from CAP cell culture supernatant. To exclude the majority of particles such as microvesicles and apoptotic bodies and to enrich mainly exosomes, culture supernatants from various engineered CAP cell lines were passed through a 0.2 μm filter. However, small remnants of EVs other than exosomes can not be excluded. Afterwards, morphology and size of exosomes were examined by Transmission Electron Microscopy (TEM). Isolated exosomal preparations showed vesicles approximately 50–150 nm in size, surrounded by membrane bilayers, independently of cell modifications (Fig 1A). This size distribution was further confirmed using dynamic light scattering (S1 Fig).

Exosomes harbour a variety of proteins on their membrane surface. Especially tetraspanin CD9 and CD63 are enriched and indicative for their identification [37]. As the engineered CAP cell lines overexpress tetraspanin CD63 as a GFP fusion protein, cell lysates and isolated exosomes were immunoblotted for the presence of GFP and CD9, while blotting with GRP78 served as a negative control for exosomes due to its expression restricted to the endoplasmic reticulum [38]. Parental CAP cells were negative for GFP, as the faint band seen in lane 1 and 5 represents a non-specific signal since it was equally visible in lanes loaded with sample buffer only (Fig 1B, middle panel, lanes 1,5), whereas cell and exosomal lysates derived from CAP-CD63-GFP cells showed a strong GFP signal (Fig 1B, middle panel, lanes 2–4 and 6–8), verifying successful molecular engineering of CAP cells. Due to the GFP fusion to the highly and variably glycosylated CD63 protein, a broad band ranging from 50–80 kDa was observed. Remarkably, the signal for the CD63-GFP fusion protein was stronger in exosomes compared to cell lysates, indicating the enrichment of exosomes. Additionally, the exclusive detection of CD9 in exosome lysates of CAP cell lines (lower panel, lanes 5–8) supports the effective exosome enrichment. Furthermore, the absence of the ER marker GRP78 indicates that exosome concentration was performed without cellular contaminants (upper panel, lanes 5–8 vs lanes 1–4). Finally, to highlight the enrichment of CD9 and depletion of GRP78 during exosome isolation, and the overexpression of the CD63-GFP fusion protein in modified CAP cells, the Western blot of Fig 1B was quantified (Fig 1C, 1D and 1E).

Due to the expressed CD63-GFP fusion protein, engineered CAP cell exosomes could be further visualised by flow cytometry by investigating ~100000 particles per measurment. As shown in Fig 1F, parental CAP cell exosomes revealed no GFP signal, whereas exosomes derived from CAP-CD63-GFP-miRNA-493 and CAP-CD63-GFP-miRNA-744 cells were around 80% GFP positive, when equal settings were applied. GFP-negative particles reprent the expected small amount of apoptotic bodies/ extracellular vesicles, which can not be excluded with the applied isolation technique. Together, these data underscore the successful production, modification and isolation of exosomes from parental and engineered CAP cell lines.

### Successful pre-loading of overexpressed miRNAs into exosomes by CAP donor cells

**Fig 2:**
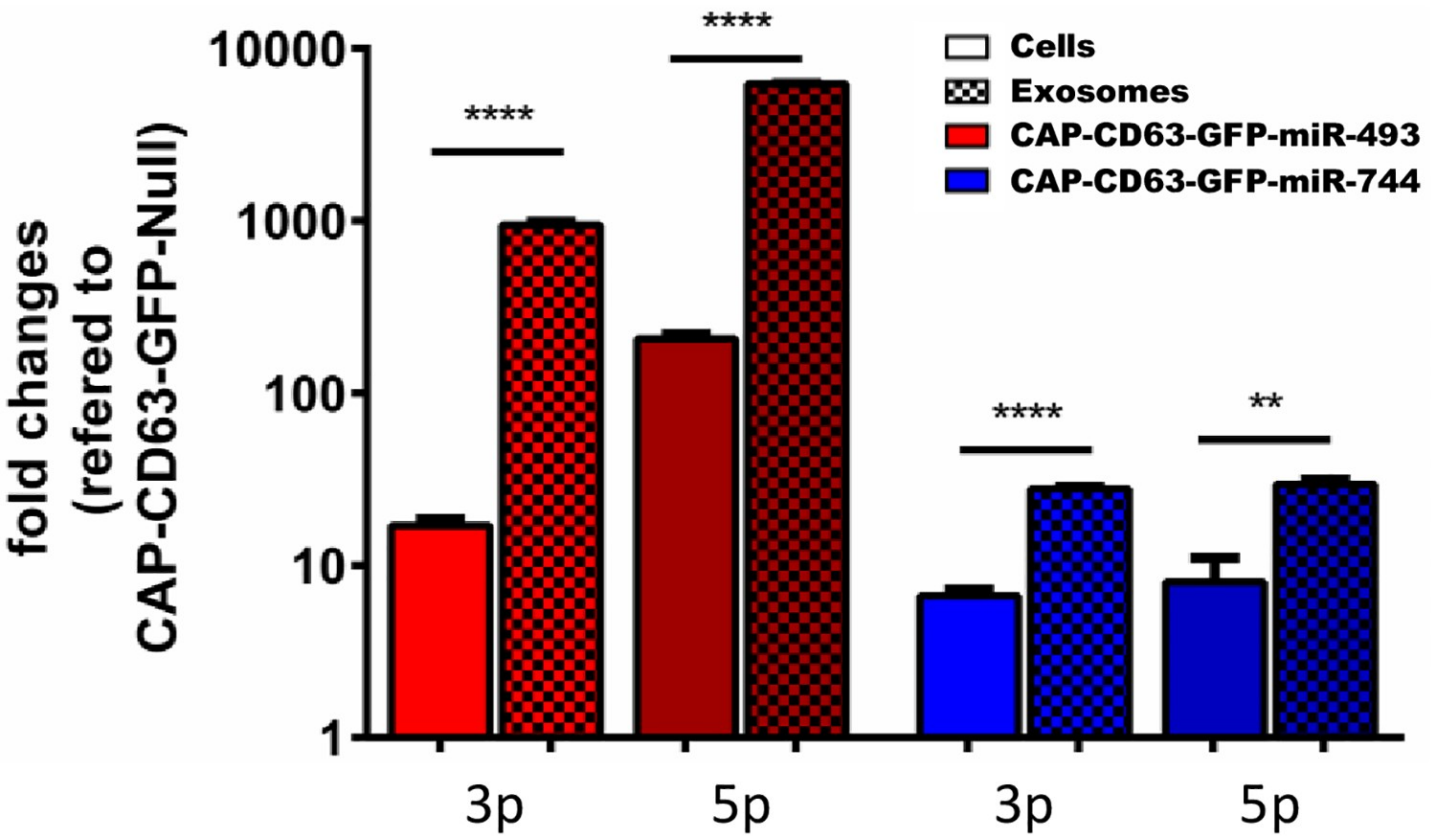
Loading of pro-apoptotic miRNA-493 and miRNA-744 into exosomes via overexpression. qPCR on overexpressed miRNAs of cellular and exosomal content. miRNA-493 shown in red bars, blue bars indicate miRNA-744 expression. miRNA overexpression in cells is given in blank bars, while checked bars represent overexpression in exosomes. CAP-CD63-GFP-Null cell line served as reference for miRNA overexpression cells, CAP-CD63-GFP-Null exosomes served as reference for exsosomes isolated from miRNA overexpression cells. U6 was used as housekeeping gene. Significance between cellular and exosomal overexpression was calculated via unpaired t-test [n = 3 replicates; mean ± SD; **p<0.01, ****p<0.0001].

To further verify miRNA overexpression in engineered CAP cells and subsequent pre-loading of these pro-apoptotic miRNAs into exosomes through cellular pathways, qPCR was performed with isolated RNA of cells and exosomes. Calculation of fold changes in relation to CAP-CD63-GFP-Null cells and exosomes after U6 normalisation for similar loading revealed an approximately 10-fold increase of the mature 3p strand and over 100-fold increase in expression of the mature 5p strand for CAP-CD63-GFP-miRNA-493 cells (Fig 2). Notably, both miRNA-493 strands reached significantly higher fold changes in exosomes than in cells of approximately 1000-fold and 10000-fold, respectively, indicating a significant exosomal enrichment. Finally, CAP-CD63-GFP-miRNA-744 cells expressed about 10-fold more of the mature 3p and 5p strand compared to the reference cell line (Fig 2). Again, a significantly stronger, 80-fold increase of miRNA-744 in exosomes derived from miRNA-744 overexpressing CAP cells was measured when compared to exosomes from reference cells. Together, the obtained data verify the overexpression of both miRNAs in engineered CAP cell lines as well as their enrichment in exosomes.

### CD63-GFP and miRNA overexpression has no impact on CAP cell growth and viability

Pre-loading of exosomes with apoptotic non-coding RNAs molecules through cellular pathways may influence the host cell line in an undesirable manner. The influence of the overexpression of miRNA-744 and miRNA-493 on CAP cell growth thereby had to be investigated. Furthermore, the overexpression of the CD63-GFP fusion protein adds additional burden to the CAP cell line and might hamper growth and viability. Therefore, viable cell density (VCD) and viability of all engineered CAP cells (Table 1) were measured during a 7 day batch culture.

**Fig 3:**
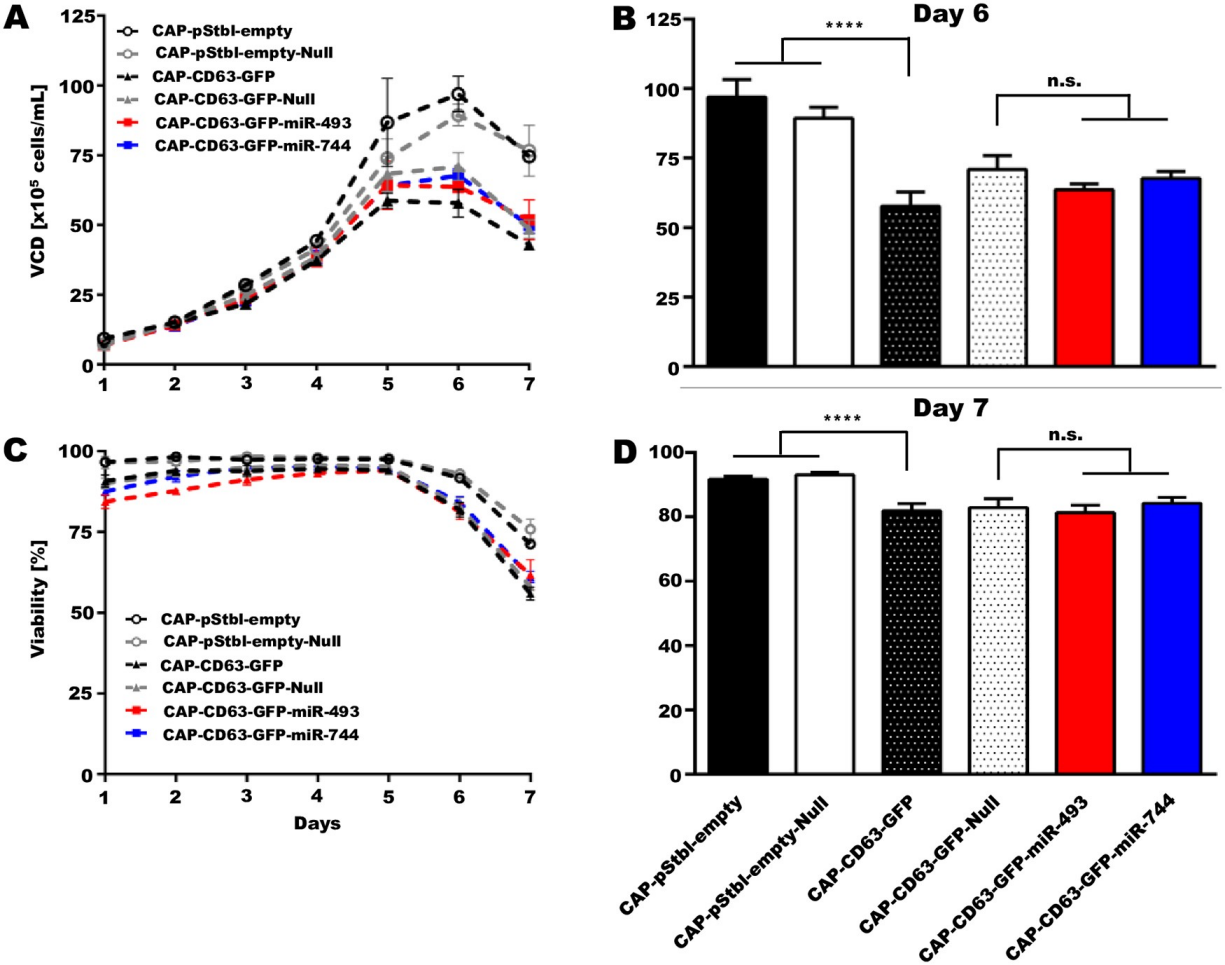
Impact of CD63-GFP and miRNA overexpression on CAP cell growth. (**A**) Viable cell density (VCD) of modified and of reference CAP cell lines during a 7 day cultivation, measured by trypan blue exclusion. (**B**) Viable cell density of modified and of reference CAP cell lines at day 6, measured via trypan blue exclusion. Significance was determined by using One-way ANOVA multiple comparison with Bonferroni correction [Each cell line cultured in 2 separate flasks; mean ± SD; n.s. = not significant, ****<0.0001]. (**C**) Viability of modified and of reference CAP cell lines during a 7 day cultivation, measured by trypan blue exclusion. (**D**) Viability of modified and of reference CAP cell llines at day 7, measured by trypan blue exclusion. Significance was determined by using One-way ANOVA multiple comparison with Bonferroni correction [Each cell line cultured in 2 separate flasks; mean ± SD; n.s. = not significant, ****<0.0001].

After 6 days, the reference cell lines not expressing the CD63-GFP fusion protein, reached a VCD of 96.9 ± 6.3 x 10^5^ and 89.4 ± 3.9 x 10^5^ cells/mL, respectively (Fig 3A). In contrast, cell lines overexpressing CD63-GFP (CAP-CD63-GFP and CAP-CD63-GFP-Null) grew at significantly lower VCDs of 57.8 ± 5.0 x 10^5^ and 70.9 ± 5.0 x 10^5^ cells/mL, respectively, putatively indicating an inhibitory effect elicited by overexpressed CD63-GFP on cell growth (Fig 3A). Surprisingly, no significant differences were observed for both pro-apoptotic miRNA overexpressing cell lines (63.6 ± 2.07 x 10^5^ cells/mL CAP-CD63-GFP-miRNA-493; 67.7 ± 2.5 x 10^5^ cells/mL CAP-CD63-GFP-miRNA-744) (Fig 3A and 3B), proving that miRNA-493 and miRNA-744 do not exert negative influence on CAP cell growth. If viability is taken into account, the control cell lines CAP-pStbl-empty and CAP-pStbl-empty-Null cells displayed a viability of 91.7 ± 0.9% and 93.0 ± 0.8%, repectively, after 6 days, whereas the CAP-CD63-GFP and CAP-CD63-GFP-Null cells showed a significantly lower viability of 81.3 ± 2.3% and 82.9 ± 2.7% (Fig 3C and 3D). Again, the viability of miRNA-493 (81.3 ± 2.4%) and miRNA-744 (84.2 ± 1.8%) overexpressing cells was comparable to that of CAP-CD63-GFP-Null reference cells (Fig 3C and 3D). Taken together, these data indicate that the overexpression of CD63-GFP negatively influences cell growth and viability. In contrast, miRNA-493 and miRNA-744 overexpression had no significant influence on CAP cell growth, albeit their previously demonstrated pro-apoptotic effects in various ovarian cancer cell lines [39,40].

### Functional delivery of pro-apoptotic miRNA-493 via engineered exosomes

**Fig 4:**
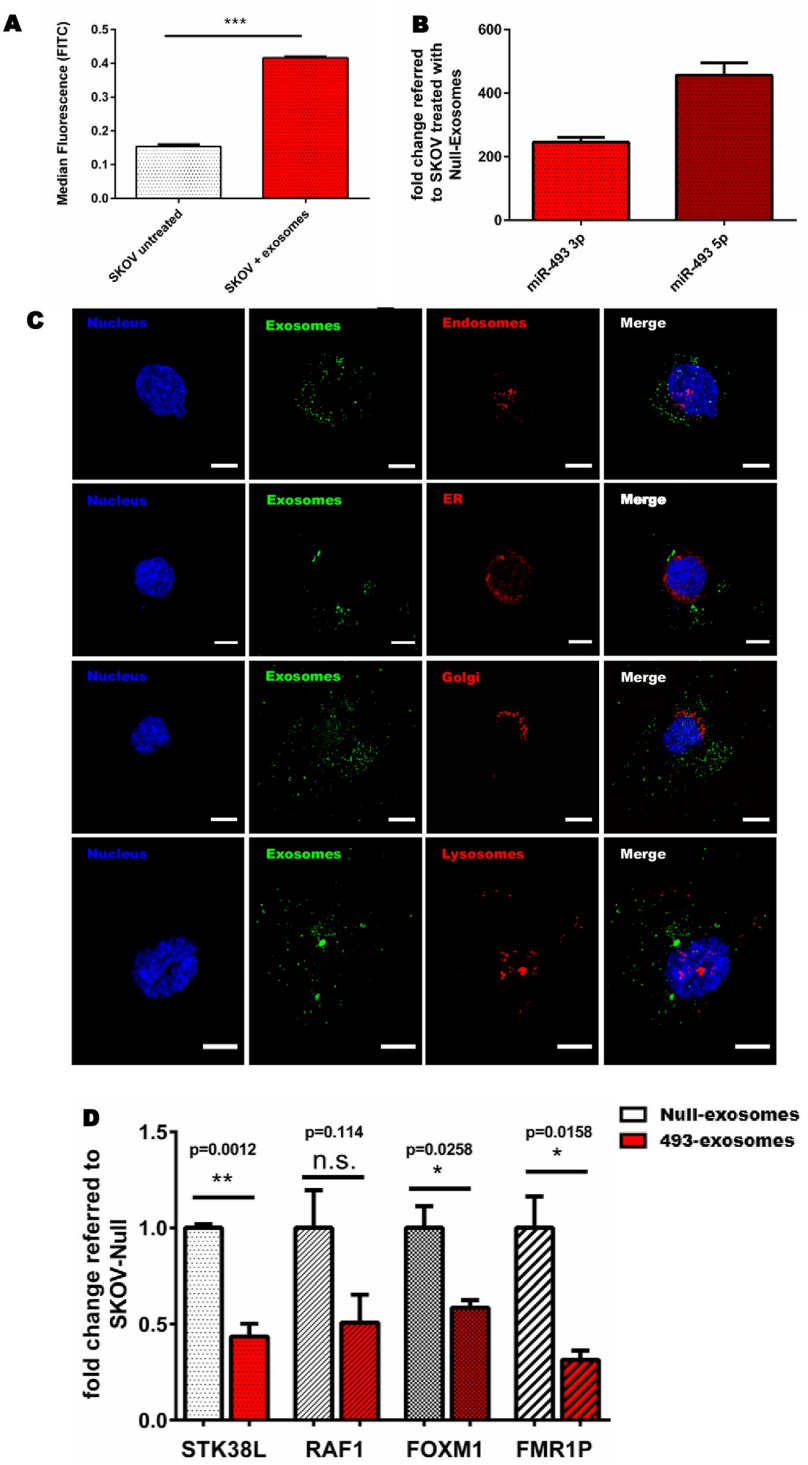
Uptake of exosomes from non-tumorigenic origin by ovarian cancer SKOV3 cells. (**A**) Flow cytometric analysis of GFP-positive SKOV3 cells after co-culturing with CD63-GFP containing exosomes. Significance was calculated employing unpaired t-test [n = 3 replicates; mean ± SD; ****p<0.0001]. (**B**) qPCR on SKOV3 cells, co-cultured with miRNA-493 pre-loaded CAP exosomes. Fold change calculations referred to miRNA-Null exosomes treated SKOV3 cells. U6 served as loading control. (**C**) Confocal microscopy images of SKOV3 cells stained for organelle marker proteins after treatment with CD63-GFP containing exosomes. DAPI (blue) indicates nucleus staining, GFP (green) represents exosomes and Alexa647 (red) shows organelle staining of endosomes, endoplasmic reticulum, golgi and lysosomes. Scale bar represents 10 μm. (**D**) qPCR on miRNA-493 target genes STK38L, RAF1, FOXM1 and FRM1P in SKOV3 cells, after exosome co-culturing for 72 h. SKOV3 cells were treated with miRNA-Null and miRNA-493 loaded exosomes all 24 h with 250 μg exosomal protein. PPIA served as loading control. Significance was determined by unpaired t-test [n = 3 replicates; mean ± SD; n.s. = not significant; *p<0.05, **p<0.01].

To verify the potential of CAP exosomes to deliver their content to recipient cells, we assessed the functionality and the effective delivery of pro-apoptotic miRNAs using exosomes isolated from CAP-CD63-GFP-miRNA-493 cells. The interaction of CD63-GFP containing exosomes with the ovarian cancer cell line SKOV3 was evaluated by flow cytometry (Fig 4A). While SKOV3 cells without exosome treatment showed a median GFP signal of 0.153 ± 0.012, treatment with CAP-CD63-GFP-miRNA-493 exosomes led to an almost 3-fold increase (0.153 ± 0.012 vs 0.417 ± 0.006) which was indicative for interaction of exosomes with target cells.

To verify not only exosome interaction but also the uptake of the non-coding RNAs delivered by CAP-CD63-GFP-miRNA-493 exosomes into receipient cells, SKOV3 cells were treated with exosomes isolated from CAP-CD63-GFP-miRNA-493 cells and CAP-CD63-GFP-Null cells and qPCR performed. As shown in Fig 4B, SKOV3 cells incubated with exosomes isolated from miRNA-493 overexpressing cells led to a about 250 and 450-fold increase of the respective miRNA-493 3p and 5p strand compared to SKOV3 cells treated with miRNA-Null exosomes. These data indicate that not only exosomes of non-tumorigenic origin interact with cancer cells but also deliver the respective miRNA-strands into the target cells.

To further verify exosome uptake into recipient cells, the intracellular localisation of the engineered exosomes was assessed by GFP expression using confocal microscopy. To determine their intracellular localization, organelles including Golgi apparatus, the endoplasmic reticulum, endosomes and lysosomes were stained with the respective organelle-specific antibodies. Confocal imaging revealed that the exosomes were mainly localised in the cytoplasm, partly close to the nucleus (Fig 4C) and no co-localization with the above mentioned organelles was observed. In addition, exosomes did not primarily fuse with or adhere to the plasma membrane of recipient cells.

Finally, we adressed the question of whether transported miRNAs are also released into the intracellular space and exert their molecular function. To this end, SKOV3 cells were treated daily with exosomal material of the cell lines CAP-CD63-GFP-miRNA-493 and CAP-CD63-GFP-Null over a period of 3 days. qPCR was performed to examine the downregulation of its reported miRNA-493 targets STK38L, RAF1, FOXM1 and FMR1P [39]. A significant downregulation of STK38L of about 56% was detected (Fig 4D). A slightly less albeit still significant suppression was observed for the genes FOXM1 and FMR1P (about 41% and 69%, respectively). The miRNA-493 target RAF1 revealed a tendential, non-significant reduced expression of around 49% (p-value = 0.114) (Fig 4D). Importantly, these data reveal that exosomes taken up by recipient cancer SKOV3 cells successfully deliver functional pro-apoptotic miRNA from non-turmorigenic origin and underscore the potential of exosomes as delivery tools for nucleic acid based pharmaceuticals.

## Discussion

Exosomes are natural carriers of various nucleic acids including mRNA, miRNA and various noncoding RNAs [41,42] and thus represent ideal vehicles for the delivery of nucleic acids. In comparison to alternative delivery systems such as nanoparticles or liposomes, exosomes are superior as they do not trigger severe immune reactions due to their natural membrane composition [25]. Additionally, exosomes efficiently deliver their content through cellular membranes and can be modified to ensure targeted transport [9,43,44]. Different immortalized cell lines have commonly been used as producers for extracellular vesicles owing to the infinite supply of cells for production, increased proliferation rates and the ease of genetic modification [31,45]. However, most immortalized cell lines originate from tumorigenic origin which eliminates their use for therapeutic exosome production since they may carry harmful or even carcinogenic constituents [46]. As an alternative, mesenchymal stem cells (MSCs) have been used for exosome production and already showed promising results in several approaches [47–49]. Nevertheless, MSCs are not ideal for exosome production, as large-scale cultivation is difficult and the final yield remains unsatisfactory [50].

In this study we evaluated the potential of the human amniocyte derived CAP cell line to be used for exosomes production. CAP cells are from human, non-tumorigenic origin and hold a fully documented history, since they were initially developed for the production of therapeutic proteins and viral vectors [32,33]. In addition, their ability to grow in suspension may greatly simplify future production and purification of exosomes at a larger scale. Taking together, CAP cells have significant advantages over currently used cell lines.

For the initial evaluation of CAP cells as production hosts for exosomes, a parental cell line was engineered to overexpress the exosome marker protein CD63 fused to GFP. This allowed for successful tracing of produced exosomes and validation of their isolation using FACS analysis, as well as tracing their delivery to recipient cells using fluorescence microscopy. Furthermore, miRNAs were overexpressed with the objective to preload therapeutic miRNAs into CAP exosomes through cellular pathways. In contrast to cellular preloading, exosomes also have been loaded with miRNAs after their isolation through electroporation [9,51]. However, this approach not only carries the risk for contamination with transfection reagents, but also shows limited transfection efficiencies [52]. Furthermore, the morphology of exosomes is altered by electroporation, leading to adverse effects such as aggregation [53]. With this study we aimed to explore, if CAP cells are capable of producing functional exosomes loaded with efficient levels of therapeutic miRNAs, which can be used for miRNA delivery to recipient cells.

Detailed characterization of CAP exosomes isolated from culture medium via several experiments verified the presence of membrane surrounded vesicles of 50–150 nm diameter, displaying the exosome marker protein CD9 as well as CD63-GFP on their membrane (Fig 1). Furthermore, these vesicles contained mature miR-744 and miR-493, which were successfully pre-loaded into exosomes by either cellular pathways or passive integration during exosome biogenesis (Fig 2). Interestingly, miRNA abundance in exosomes was significantly higher than in CAP cells, suggesting active loading mechanisms for miRNA-493 and miRNA-744. Several nucleic acid sequences such as GGAC, GGCC, GCCU, CCCG or UGCC can support active incorporation of miRNAs into exosomes and are therefore classified as EXOmiRNA motives [30,54,55]. Some of these motives are indeed present in the 3p strand of miRNA-493 and miRNA-744, which may leads to preferential loading into exosomes (S2 Fig). These data indicate that CAP cells are not just able to produce exosomes but are also capable of efficiently loading those exosomes with therapeutic miRNAs.

MiRNA-493 and miRNA-744 are known to execute pro-apoptotic effects in several cancer cell lines [39,40]. While, miRNA-493 promotes apoptosis in liver cancer and suppresses tumorigenesis in hepatocellular carcinoma [56,57], miRNA-744 is reported to induce apoptosis in ovarian and cervical carcinoma [40,58], but is contradictory known to enhance cancer progression and metastasis in laryngeal squamous cell carcinoma [59]. Due to these impacts on cell growth, negative effects of their overexpression on the CAP host cells may limit the potential of exosomal pre-loading strategies. However, neither miRNA-493 nor miRNA-744 inhibited CAP cell growth, although both miRNAs were heavily overexpressed (Fig 3). These results indicated a possible resistance of CAP cells to the overexpression of pro-apoptotic miRNAs. Human amniocytes, where CAP cells were originally derived from, have previously been shown to possess stem-cell-like characteristics and express canonical regulators associated with pluripotency and stem cell repression [60], which may render them with a higher resistance to inducers of apoptosis [61,62]. Alternatively, relevant target genes of pro-apoptotic miRNAs in tumor cells may be absent in amniocytes of non-tumorigenic origin and thereby enable overexpression in an efficient manner. The cell line-specific mechanism of miRNAs, has already been observed for miRNA-744, which showed both pro-apoptotic and anti-apoptotic effects in two different tumorigenic cell lines [59,63]. It is therefore not surprising that miRNA-493 and miRNA-744 exhibit different effects in cell lines derived from amniocytes and ovarian cancer, respectively. Importantly, this cellular property of resistance to some pro-apoptotic miRNAs may represent an advantage for the production process of exosomes containing therapeutic molecules and render CAP cells as an ideal host for exosome production.

After accessing efficient production and loading of CAP exosomes with therapeutic miRNAs, their functionality was investigated. Exosomes interact with target cells via membrane fusion, endocytosis or interaction with membrane receptors [8,64,65]. In this study, co-cultivation experiments of modified CAP exosomes and SKOV3 cells, where miRNA-493 and miRNA-744 showed pro-apoptotic effects previously [39,40], revealed an interaction between exosomes and recipient cells and confocal microscopy showed an intracellular uptake of CAP exosomes by SKOV3 cells (Fig 4). However, no co-localization with endosomes or lysosomes was observed, which corresponds to similar findings describing exosomes uptake mainly by endocytosis into the cytoplasm using exosomes from tumorigenic origin [65,66]. Next, we demonstrated that exosomes taken up by endocytosis were capable of functionally releasing their loaded miRNA by downregulation of several known miRNA-493 target genes including STK38L, FOXM1 or FMR1P. Since miRNA regulation of these target genes was reported to induce apoptosis in ovarian cancer cells, their downregulation after exosomal delivery of miRNA-493 verifies the functionality and potency of exosomes to transport therapeutic molecules to target cells. In addition, RAF1, which is also known as the target gene of miRNA-493, shows a clear trend towards downregulation, however, significane was not reached. In contrast to our study, Kleemann et al., 2019 validated this target using miR-493 mimics transfected in artificially high amounts by lipofection into target cells. These high levels were probably not achieved using cellular loading of exosomes [39]. Nethertheless, the exact mechanisms of exosomal miRNA loading and release are not fully understood and require further investigation.

In summary, this study describes human amniocyte derived CAP cells as a novel source for engineered exosomes of non-tumorigenic origin. Furthermore, it was demonstrated that pro-apoptotic miRNAs could be pre-loaded via cellular pathways into exosomes. Most importantly, exosomes produced by CAP cells delivered functional pro-apoptotic miRNAs to receptor cells, supporting their potential to produce exosomal delivery vehicles for therapeutics. By revealing CAP cells as a novel and efficient cell line for exosome production, the pharmaceutical use of exosomes could become strikingly more feasible.

## Methods

### Cell culture

CAP (CEVEC‘s Amniocyte Production) cells were cultured in animal component free CAP-CDM medium (Merck, Darmstadt, Germany), supplemented with 6 mM ultraglutamine (Lonza, Basel, Switzerland) and 50 μg/L Long R^3^ IGF-I (Sigma–Aldrich, München, Germany) at 140 rpm (25 mm orbit) with 5% CO_2_ and 85% humidity at 37 °C. Passaging to a maximum of 20 passages was performed every 3–4 days to a denstiy of 0.5 x 10^6^ cells/mL and viable cell density and viability were determined via trypan blue exclusion using CEDEX XS (Roche Diagnostics, Mannheim, Germany). CAP cell pools stably overexpressing CD63-GFP were generated using the pStbl-bsd-CMV and pCMV6-AC-GFP vectors (OriGene Technologies Inc., Rockville, MD, USA) and selected using 5 μg/mL blasticidin (Invitrogen, Carlsbad, USA). CAP pools stably overexpressing miRNA-493 or miRNA-744 were generated using the miRNASelect^™^ pEGP-miR-Cloning and Expression vector and selected by 2 μg/mL puromycin (Invitrogen, Carlsbad, USA). The resulting cell pools were specified as CAP-CD63-GFP-miRNA-493, CAP-CD63-GFP-miRNA-744, CAP-CD63-GFP-miRNA-Null, CAP-empty-Null. For growth evaluation of engineered CAP-cells, they were seeded in a working volume of 50 ml with a density of 0.5 x10^6^ cells/ml. Viable cell density and viability were measured over a period of 7 days. SKOV3 cells were cultured in DMEM with 10% fetal calf serum at 5% CO_2_ and 85% humidity at 37 °C.

### Enrichment of exosomes

Exosomes were enriched from CAP cell culture supernatant after a 3–4 day cultivation with VCD > 5x10^6^ cells/ml. Cell suspension was centrifuged for 15 min at RT with 5000 x *g* to pellet the cells. Afterwards, the supernatant was 0.2 μm filtrated and mixed 1:3 with a 36% PEG6000 solution. After an overnight incubation at 4 °C, the mixture was centrifuged at 10000 x *g* at 4 °C for 1 h to pellet the exosomes. Exosome pellets were resuspended in PBS, RIPA-buffer, media or Trizol depending on further experiments. Due to the lack of a suitable device to quantify isolated exosomes, the amount of exosomes was determined for subsequent experiments via BCA assay. Exosomes were stored for 2–3 days at 4 °C, while for long term storage they were frozen at -20 °C.

### Electron microscopy

Exosomes were prepared as described by Walther and Ziegler (2002) with minor modifications. Samples were high pressure frozen, freeze substituted and embedded in Epon. Ultrathin sections were cut with an ultramicrotome and visualised with a Jeol 1400 transmission electron microscope (Jeol Inc.) [67].

### RNA isolation

RNA was isolated using the miRNeasy Kit (Qiagen, Hilden Germany) according to the manufacturer’s instructions. Also a miRNA enriched fraction was isolated by performing the specific instructions for short RNA molecules (>200 nt) as described in the miRNeasy handbook. Isolated miRNA was analysed for purity and concentration using a Nanodrop^™^ 1000 Spectrophotometer for measuring absorbance at 260 nm (Thermo Fisher Scientific, Darmstadt, Germany).

### Quantitative real-time qPCR

miRNA analysis was performed using the miRCURY LNA^™^ kit (Qiagen) according to the manufacturer’s instructions. The following miRCURY LNA miRNA qPCR assays (Qiagen) were applied: hsa-miRNA-493-3p; hsa-miRNA-493-5p; hsa-miRNA-744-3p; hsa-miRNA-755-5p and U6 snRNA (hsa, mmu) which served as housekeeping gene. Assays were performed in triplicates and data were recorded using the LightCycler^®^ 480 Instrument II (Roche Diagnostics). Target mRNA expression was evaluated using GreenMasterMix (Gennaxxon Bioscience, Ulm, Germany). Primers for qPCR were FMR1P_FW (5′-AATCCAAAAGAACAGTGGCATT-3′), FMR1P_RV (5′-GGAATCCCAGAAACCTGA ACT-3′), FOXM1_FW (5′-CCACTGGATGTTGGATAGGC-3′), FOXM1_RV (5′-AGA AACGGGAGACCTGTGC-3′), RAF1_FW (5′-TGGGAAATAGAAGCCAGTGAA-3′), RAF1_RV (5′-CCTTTAGGATCTTTACTGCAACATC-3′), STK38L_FW (5′-CAAAGA CCACCAGTCACACAA-3′), STK38L_RV (5′-GAAGAAGAACAGGAGACAACTGG-3′) and PPIA_FW (5′-ATGCTGGACCCAACACAAAT-3′), PPIA_RV (5′-TCTTTCACTTTG CCAAACACC-3′) which served as housekeeping gene.

### Exosomal uptake

For the evaluation of exosomal uptake, 3x10^5^ SKOV3 cells were seeded into a 6 well plate in 5 mL DMEM + 10% FCS (Greiner bio-one, Frickenhausen, Germany). CD63-GFP containing exosomes were quantified using BCA-assay according to Walker (1994) [68] and 150 μg exosomal protein were incubated with seeded SKOV3 cells for 24 h at 37 °C. Exosomal uptake was assesed by measuring median FITC-fluoresence of treated and untreated SKOV3 cells. For the evaluation of functional exosome uptake, 5x10^5^ SKOV3 cells were seeded into a 25 cm^2^ flask and 250 μg exosomal material was added daily over a period of 3 days. Delivery of functional miRNAs was investigated by performing qPCR on miRNA-493 target genes.

### Flow cytometry analysis

Exosomes were analysed by flow cytometry using the MACSQuant^**®**^ Analyzer 10 (Miltenyi Biotec, Bergisch Gladbach, Germany). CD63-GFP containing exosomes were measured directly after isolation without further staining. The uptake of CD63-GFP containing exosomes by SKOV3 cells was quantified using flow cytometric analysis. Therefore, 5x10^5^ SKOV3 cells were washed two times with PBS and applied for flow cytometry. Resulting data was analysed using the MACSQuantify^™^ Software.

### Immunoblotting

Cell and exosomes were lysed in RIPA buffer (1% v/v Nonidet^™^ P40, 0.5% w/v sodium deoxycholate, ethylenediaminetetraacetic acid 1mM, all agents Carl Roth, Karlsruhe, Germany). Protein concentrations of cell and exosomal lysates were determined using BCA-assay according to Walker (1994) [68]. For the separation of 35 μg protein in a 25 μL volume a sodium dodecyl sulfate polyacrylamide gel electrophoresis was performed using TruPAGE^™^ Precast 4–20% gradient gels (Sigma-Aldrich). After the separation of proteins and transfer on a polyvinylidene fluoride membrane by blotting for 75 min with 60 mA/gel, immunoblotting with the following antibodies was performed over night at 4 °C: Anti-GFP78 BiP antibody (Abcam, Cambridge, UK, #21685; 1:1000); Anti-turbo-GFP polyclonal antibody (Evrogen, Moscow, Russia, #AB513; 1:1000) and Anti-CD9 monoclonal antibody (Thermo Fisher Scientific #10626D, 1:500). The incubation with the respective secondary antibody (anti-mouse IgG- HRP (Sigma Aldrich #A4416; 1:5000), anti-rabbit-IgG-HRP (Cell Signaling Technology, Frankfurt, Germany, #cs7074; 1:5000) was at RT for 1 h and the signal was detected using the Fusion FX imager (Vilber Lourmat, Eberhardzell, Germany).

### Fluorescence microscopy

In order to verify exosomal uptake by recipient cells and the intracellular localization, confocal images were recorded. For this, 15x10^3^ SKOV3 cells were seeded into Nunc^™^ Lab-Tek^™^ II Chamber Slides^™^ (Nunc, Roskilde, Denmark) and cultured with exosomes overexpressing CD63-GFP. After 24 h, SKOV3 cells were fixed with 4% PFA and permeabilised using 0.1% TritonX. Afterwards cells were blocked with a 3% BSA, 0.1% Tween20 and 0.3 M Glycin solution. Organelles were stained with the specific antibodies Anti-EEA1 (Abcam #ab2900; 1:200), Anti-Lamp1 (Abcam #ab24170; 1:200), Anti-GM130 (BD BioScience, Heidelberg, Germany, #610822; 1:200) and Anti-GRP78 (Abcam #21685; 1:300). As secondary antibodies Anti-Rabbit AlexaFluor594 (Jackon ImmunoResearch, Cambridge, UK, #711-585-152; 1:500) and Anti-mouse Rhodamine Red-X (Jackson ImmunoResearch #115-295-206; 1:100) were used. Isotype controls were purified mouse IgG1 ĸ isotype control (BD BioScience #554121; 1:200) and rabbit IgG, polyclonal isotype control (Abcam #ab37415; 1:40). Cells were mounted with Prolong^®^ Diamond Antifade Mountant medium (Invitrogen, Darmstadt, Germany) and covered with a glass slide. Images were recorded with the confocal microscope Zeiss LSM 710 (Carl Zeiss, Oberkochen, Germany).

### Statistical analysis

Statistical differences between generated data were determined using GraphPad Prism 6 software. ANOVA test with Bonferroni correction or Student’s ***t***-test was applied to calculate statistical differences of mean between groups. All mean values of groups during the report are given as mean ± SD.

## Supporting information

S1 Fig Size distribution of isolated exosomes from modified CAP cell lines measured via dynamic light scattering. (TIF)

S2 Fig Stem-loop sequence of miRNA-494 and miRNA-744. 3p and 5p strands are highlighted in yellow, EXO motifs are marked in red.
(TIF)

S3 Fig Loading of pro-productive miRNA-30a into exosomes via overexpression in CAP cells. (A) Size distribution of isolated exosomes from miRNA-30a overexpressing CAP cells measured via dynamic light scattering. (B) GFP-positive exosomes isolated from miRNA-30a overexpression CAP cells measured via flow cytometry. (C) miRNA-30a overexpression in CAP cells and exosomes. (D) Growth behavior of miRNA-30a overexpression CAP cells. (E) Viability of CAP cells overexpressing miRNA-30a.
(TIF)
