# Supplementary figures and images for: Human CAP cells represent a novel source for functional, miRNA-loaded exosome production
Source: PLoS One. 2019 Aug 28;14(8):e0221679. doi: 10.1371/journal.pone.0221679 (PMC6713437; doi:10.1371/journal.pone.0221679)

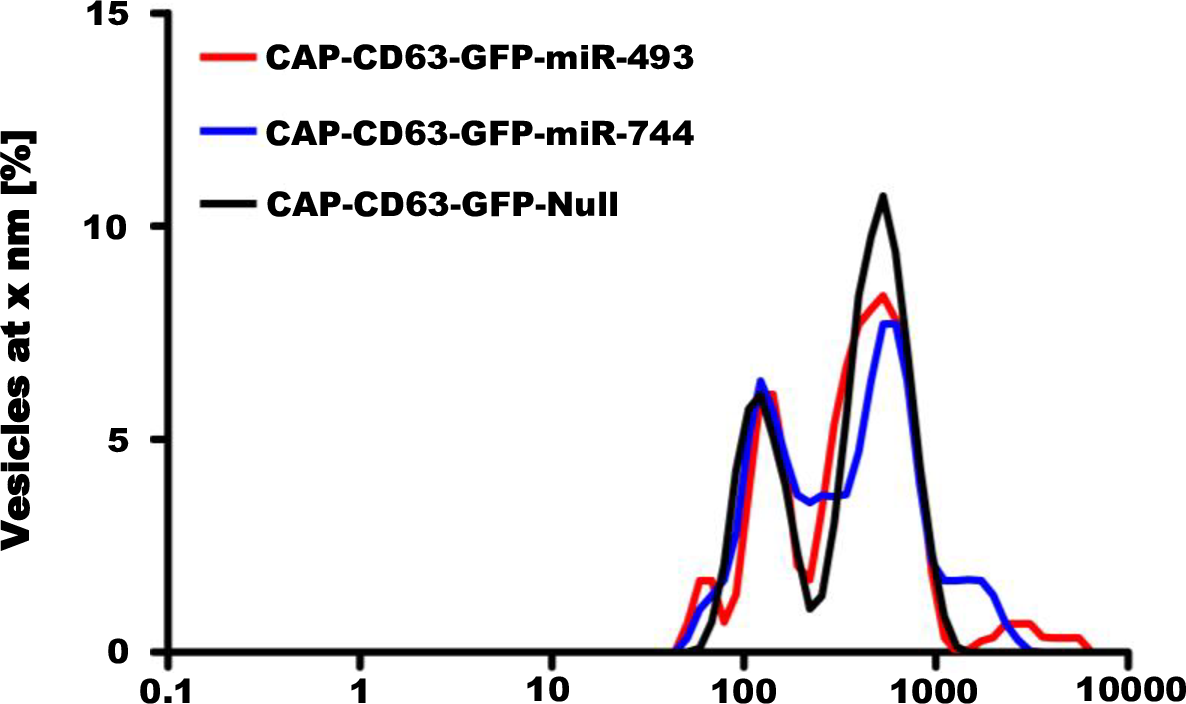

Supplement: S1 Fig — (TIF) [file pone.0221679.s001.tif]

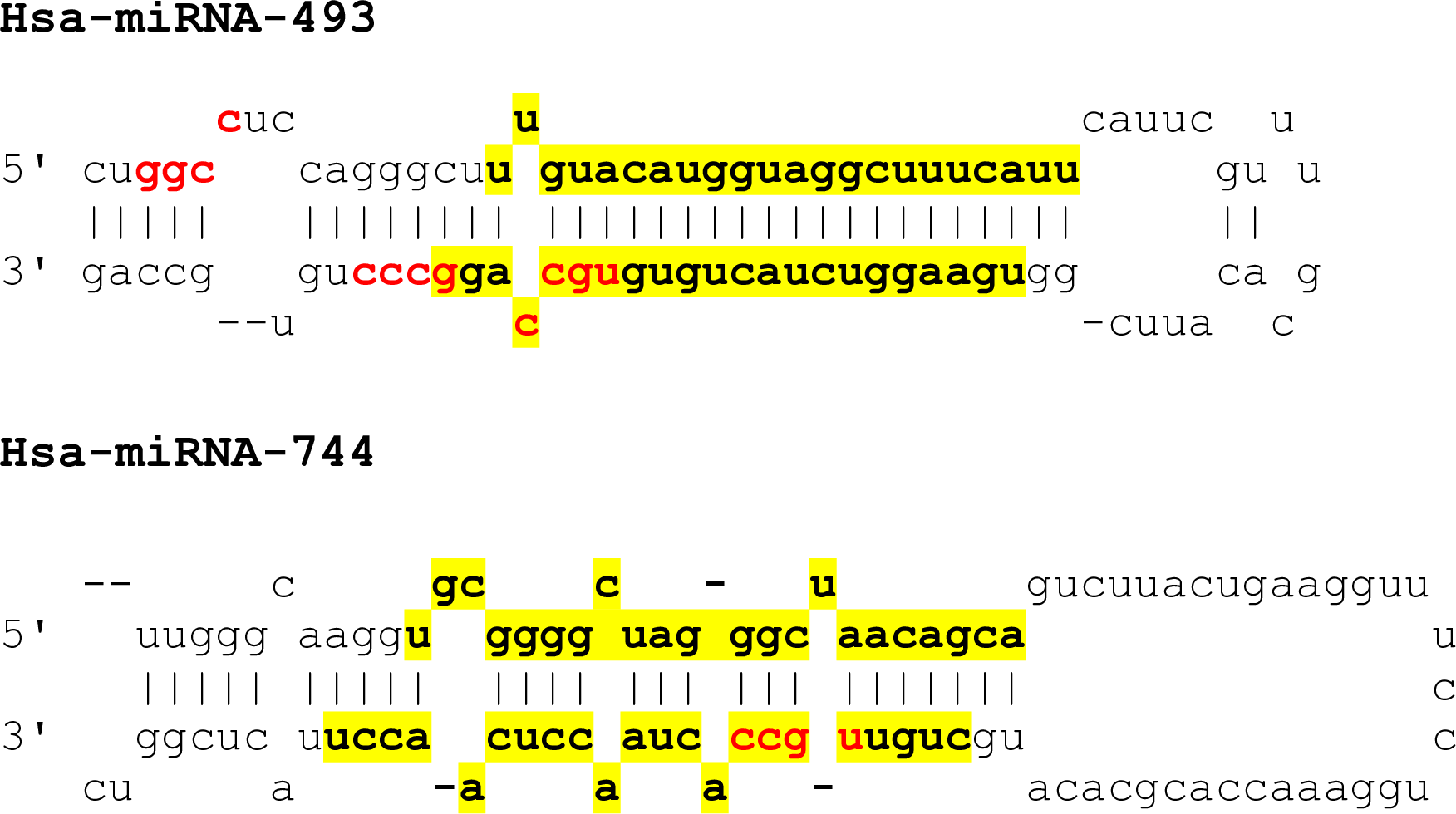

Supplement: S2 Fig — 3p and 5p strands are highlighted in yellow, EXO motifs are marked in red. (TIF) [file pone.0221679.s002.tif]

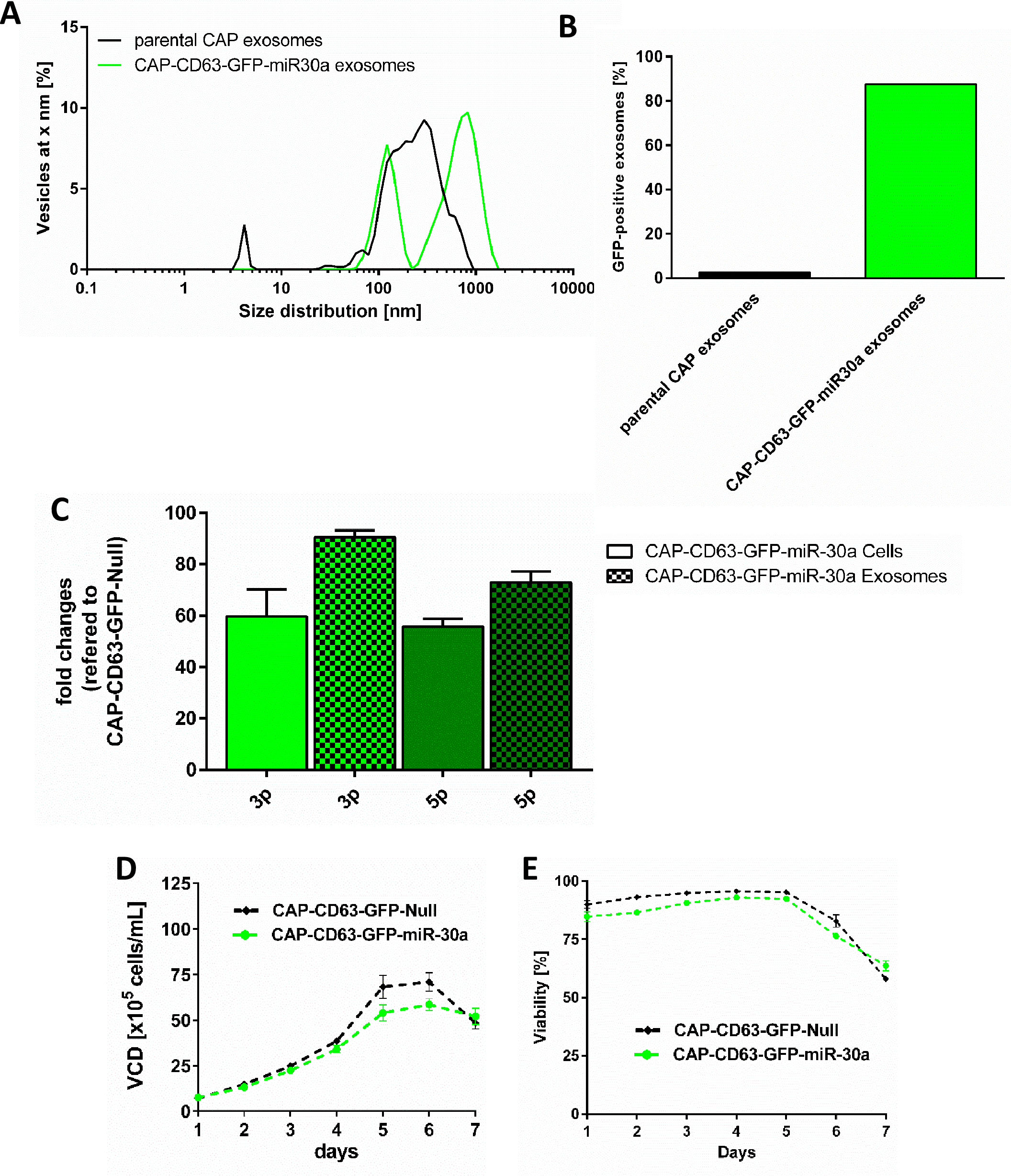

Supplement: S3 Fig — (A) Size distribution of isolated exosomes from miRNA-30a overexpressing CAP cells measured via dynamic light scattering. (B) GFP-positive exosomes isolated from miRNA-30a overexpression CAP cells measured via flow cytometry. (C) miRNA-30a overexpression in CAP cells and exosomes. (D) Growth behavior of miRNA-30a overexpression CAP cells. (E) Viability of CAP cells overexpressing miRNA-30a. (TIF) [file pone.0221679.s003.tif]
